# Effects of Longitudinal External Magnetic Field on Metal Transfer Behavior and Spatter Formation in CO_2_ Arc Welding

**DOI:** 10.3390/ma18030537

**Published:** 2025-01-24

**Authors:** Dang Khoi Le, Shinichi Tashiro, Bin Xu, Anthony B. Murphy, Quang Ngoc Trinh, Van Hanh Bui, Toshifumi Yuji, Sarizam B. Mamat, Kenta Yamanaka, Manabu Tanaka, Lei Xiao

**Affiliations:** 1 Joining and Welding Research Institute, Osaka University, Osaka 567-0047, Japan; khoi.ld.hust@gmail.com (D.K.L.); xubin2019@bjut.edu.cn (B.X.); sarizam@umk.edu.my (S.B.M.); tanaka.manabu.hq@osaka-u.ac.jp (M.T.); 2 Engineering Research Center of Advanced Manufacturing Technology for Automotive Components, Beijing University of Technology, Beijing 100124, China; 3 CSIRO, Manufacturing, Lindfield, NSW 2070, Australia; tony.murphy@csiro.au; 4 School of Mechanical Engineering, Hanoi University of Science and Technology, Hanoi 100-000, Vietnam; hanh.buivan@hust.edu.vn; 5 Faculty of Education, University of Miyazaki, Miyazaki 889-2192, Japan; yuji@cc.miyazaki-u.ac.jp; 6 Faculty of Bioengineering and Technology, University Malaysia Kelantan, Jeli Campus, Jeli 17600, Malaysia; 7 Institute for Materials Research, Tohoku University, 2-1-1 Katahira, Aoba-ku, Sendai 980-8577, Japan; kenta.yamanaka.c5@tohoku.ac.jp; 8 School of Materials Science and Engineering, Lanzhou Jiaotong University, Lanzhou 730070, China

**Keywords:** CO_2_ arc welding, longitudinal magnetic field, repelled transfer, spatter formation

## Abstract

Excessive spatter formation in conventional CO_2_ arc welding significantly diminishes welding quality and efficiency, posing a critical challenge for industrial applications. To address this issue, this study investigated the mechanisms of metal transfer behavior and spatter formation under the influence of a longitudinal magnetic field (LMF) using a shadow-graph technique with high-speed imaging and back-laser illumination, also coupled with Computational Fluid Dynamics (CFD)-based arc-droplet numerical simulations. The results show that increasing the magnetic flux density (MFD) from 0 to 2 mT shifted the transfer mode from the repelled transfer to the globular transfer, while higher MFDs (3–4 mT) induced rotating repelled transfer. The globular transfer at 2 mT was considered to be primarily produced by the centrifugal effect due to the rotational motion of the molten metal inside the droplet, which was caused by the Lorentz force affected by LMF. The higher droplet temperature in this condition also contributed to forming this transfer mode, preventing the formation of repelled transfer through a decrease in the arc pressure. On the contrary, in the higher MFDs, the droplet temperature decreased to increase the arc pressure, lifting the droplet up. Furthermore, the very strong centrifugal effect rotated the molten metal column around the wire axis to induce the rotating repelled transfer. The spatter formation was found to occur with the two-stage motion of the curved long tail without LMF and at 4 mT, and also with the exploding molten metal column at 4 mT, due to an imbalance of the Lorentz force acting on the molten metal. On the other hand, the neck formation facilitated smooth droplet detachment without forming the curved long tail at 2 mT, reducing spatter significantly. These findings offer valuable insights for optimizing welding quality and efficiency by stabilizing globular transfer under an optimal LMF.

## 1. Introduction

Arc welding is a mechanical manufacturing process that creates a permanent joint, playing an important role in industrial applications. The process begins when an electric arc is formed between two electrodes, one being the base metal. The arc is ignited by a short circuit contact at points of extremely high current density on the uneven surfaces of the anode and cathode. The arc generates high energy flux, melting the base metal and forming a weld pool [1]. Among various arc welding processes, gas metal arc welding (GMAW) is widely utilized for semi-automatic and automatic welding works, and therefore is studied extensively via both numerical simulations and experimental observation to improve our understanding of the process mechanism. In GMAW, pure CO_2_ shielding gas is widely utilized. This technique is known as CO_2_ arc welding and stands out for its simplicity, high productivity, and cost-effectiveness, making it a valuable process for industrial applications [2]. However, its broader adoption is hindered by challenges at high-current welding conditions, where poor weld surface quality and excessive spatter often occur [3,4]. To address these limitations, for example, CO_2_ arc welding with a pulsed rectangular current waveform has been developed to achieve better control of metal transfer behavior and reduce spatter generation. Under optimized settings—peak currents of 450–550 A and pulse frequencies of 450–750 Hz—the process achieves more consistent droplet detachment and smaller droplet sizes compared to conventional CO_2_ arc welding. This advancement reduces total spatter volume by 70%, with a particularly notable decrease in large-diameter spatter [2,5]. Although the pulse current can address the issue of spatter generation, the welding power source tends to become expensive. Consequently, alternative solutions need to be considered. As modern industry demands increasingly high standards, interest in optimizing conventional techniques is on the rise. One promising option is the application of an external magnetic field (EMF) in the welding process. This study aims to explore the effects of applying an EMF on the CO_2_ arc welding, providing new insights into its impact on welding outcomes.

EMF in welding refers to a magnetic field that is either intentionally applied or naturally present in the welding environment but originates outside the original welding system [6,7]. The intentional EMF can be generated using permanent magnets or electromagnets, which are positioned close enough to influence the welding process. When electromagnets are used, the EMF can be classified as either a direct current (DC) or an alternating current (AC). Furthermore, electromagnets are classified into sub-groups based on their coil designs and arrangements: Transverse Magnetic Field (TMF), longitudinal magnetic field (LMF), or Cusp Magnetic Field (CMF), each with distinct characteristics and applications in welding. EMFs significantly influence the welding process by shaping the arc, affecting the metal transfer, and improving the welding bead formation. They alter arc behavior to influence the droplet detachment and the dynamics of the weld pool, thereby improving the weld seam quality and joint performance. The application of EMF helps minimize defects and optimize the welding process [6,7]. This study focuses on the effects of a DC LMF on the metal transfer behavior in CO_2_ arc welding, paying particular attention to the phenomenon of repelled transfer.

Tsao et al. developed a mathematical model for GMA welding to simulate the electromagnetic force, velocity, and temperature fields in the weld pool, accounting for transient effects, moving boundaries, and force interactions [8]. Their findings highlight that high-frequency spray transfer of molten droplets, combined with Lorentz force effects, significantly increases weld pool depth, with higher frequencies producing deeper welds. Chang et al. utilized a LabView-based system to synchronize arc images, molten metal dynamics, and electrical signals (welding current and voltage) during short-circuit GMAW with pulsed LMF [9]. Their results showed that combining a low-frequency magnetic field during the early arc-burning phase with a high-frequency magnetic field during the short-circuit stage enhances metal transfer frequency and reduces spatter by effectively controlling the forces acting on the droplet. LMF changes the arc current and current density distribution in the arc, compressing it and increasing energy density [7]. Chang et al. further analyzed LMF’s effects on the arc shape in CO_2_ arc welding through mathematical modeling based on experimental and theoretical insights [10]. Under the influence of LMF, the upper arc constricts while the lower arc extends, creating a bell-shaped profile that rotates at high speed within an optimal range of magnetic field intensity. This enhances the arc’s maximal temperature, maximal current density, and voltage [11]. Applying EMF in CO_2_ arc welding causes the arc to contract and increases the heat transfer area, improving the heat distribution and liquid metal flow, which positively impacts spatter rates and weld quality. Additionally, an increasing excitation current enhances thermal ionization, leading to faster plasma motion toward the cathode and changes in the arc’s pressure field, which ultimately affects the arc temperature, current density, and the plasma’s spiral motion [12,13,14]. EMFs, including LMF, improve arc flow velocity and static pressure as the arc’s angular velocity rises under electromagnetic force, regardless of the magnetic field frequency. A low-frequency magnetic field alters droplet rotation in high-current GMAW, while a high-frequency field enhances the inertia of molten metal [15]. While most studies focus on the effects of EMFs on short-circuit, globular, or spray transfer, there are various metal transfer modes in GMAW that remain less explored. Additionally, there is almost no research on the mechanism of spatter formation in CO_2_ arc welding with EMF application, even though similar research on conventional CO_2_ arc welding has been performed. Ersoy et al. observed the spatter generation in GMAW [16]. It indicated that arc start instability in GMAW leads to higher spatter generation. Cai et al. estimated the spatter rate in GMAW-S based on Partial Least Square Regression (PLSR) [17]. They reported that different spatter rates, ranging from 1.02% to 5.80%, can be caused by various disturbances, such as changes in welding voltage, material surface, wire feeding speed, and shielding gas. Some papers also reported that controlling the current waveform by using a pulsed welding power source can reduce the spatter formation as well [18,19,20,21]. Meanwhile, Xue et al. indicated that Direct Current Electrode-Negative (DCEN) GMAW causes less spatter than Direct Current Electrode-Positive (DCEP) GMAW [22]. Notably, LMF can adjust arc and droplet inclination angles, which is especially advantageous in repelled transfer. The mechanism by which LMF influences spatter reduction through repelled transfer in CO_2_ arc welding remains an unexplored area in existing studies. Clarification of this mechanism is very important for advancing our understanding of the CO_2_ arc welding process.

This study investigates the effects of LMF on metal transfer behavior and spatter formation during CO_2_ arc welding. Bead-on-plate welding experiments are conducted using a commercial solid wire, with a hollow magnet exciting coil generating the external magnetic field. The magnetic flux density (MFD) is controlled by adjusting the coil’s excitation current. To analyze the metal transfer behavior, the shadowgraph technique is employed, utilizing a high-speed video camera (HSVC) to capture the image. Additionally, a numerical simulation model is developed to complement the experimental findings, providing insights into the arc and droplet, including the fields of temperature, flow velocity, and driving forces acting on the droplet. The proposed approach offers a significant advantage by extending the optimal welding current range for achieving stable CO_2_ arc welding with solid wire. This contribution is particularly valuable for industrial applications, where enhancing the stability and efficiency of such processes is crucial. Despite its potential, this approach remains a knowledge gap in the field, warranting further investigation.

## 2. Materials and Methods

### 2.1. Materials and Welding Conditions

The bead-on-plate welding experiments were conducted using mild steel plates (SS400—JIS G 3101) with dimensions of 300 mm × 50 mm × 9 mm. A commercial mild steel solid wire (JIS Z3312 YGW11) with a 1.2 mm diameter, classified as AWS A5.18 ER70S-G, was used as a filler material. Table 1 details the composition of base metal and filler metal.

An external hollow magnet exciting coil was employed to generate the magnetic field, positioned to encompass the welding torch nozzle. The MFD, varying from 0 to 4 mT, was produced using a DC power source.

The welding power source (DP-350, OTC Daihen, Kobe, Japan) was operated in Direct Current Electrode-Positive (DCEP) mode and equipped with a wire feeder system for the welding process. A medium welding current of 250 A was applied. The flow rate of CO_2_ shielding gas was maintained at 20 L·min^−1^. The welding voltage varied from 33.5 V to 34.0 V to maintain a constant arc length of 3 mm. The Contact-Tip-to-Work Distance (CTWD) of 20 mm was fixed during the experiments. The wire extension was consistently maintained at 10 mm above the plate’s surface prior to welding. An actuator moved the plate at a constant velocity of 5 mm·s^−1^. A summary of the experimental conditions is provided in Table 2.

### 2.2. Metal Transfer Observation

This experiment compared metal transfer behavior and spatter formation in CO_2_ arc welding with and without the application of LMF. Observations were carried out using a HSVC (Memrecam Q1v, Nac Image Technology, Minato City, Japan), a 640 nm wavelength laser illumination system (Cavilux HF System, Cavitar, Tampere, Finland), and an objective lens with a 200 mm focal length and a 1/4 focus ratio.

**Figure 1:**
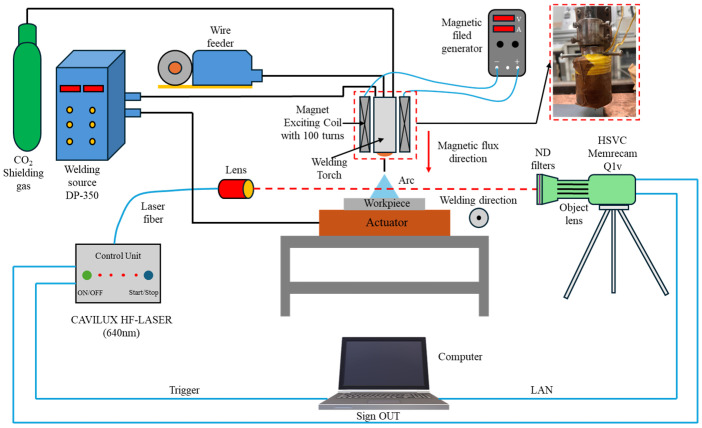
Schematic illustration of metal transfer behavior observation.

The metal transfer behavior was recorded at a frame rate of 4000 fps, with an aperture setting of f/5.6 and an exposure time of 26 μs. To minimize the intense arc radiation, which would otherwise obscure the metal transfer observation, six Neutral-Density (ND) filters—five ND-8 filters and one ND-4 filter—were utilized. Figure 1 presents the experimental setup used to record the metal transfer behavior.

## 3. Results

### 3.1. Experimental Results

#### 3.1.1. Metal Transfer Behavior

**Figure 2:**
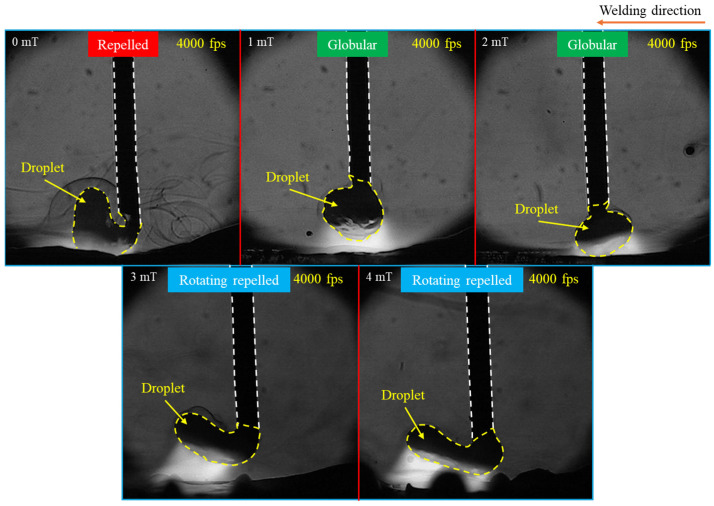
Typical images of metal transfer behavior under different MFDs.

Figure 2 shows typical images of metal transfer behavior under different MFDs obtained through HSVC, revealing significant changes in metal transfer modes. The repelled transfer mode—where a big droplet was pushed upward—was observed without LMF, while the transfer mode with LMF transitioned to globular transfer at MFDs of 1 mT and 2 mT and to a rotating repelled transfer mode—where the droplet was elongated, pushed upwards, and rotated around the wire—at 3 mT and 4 mT. For clarity, the data on metal transfer behavior in conventional CO_2_ arc welding and under the MFDs of 2 mT and 4 mT were selected for analysis, as these cases exhibited more distinct metal transfer behavior compared to the conditions at 1 mT and 3 mT.

**Figure 3:**
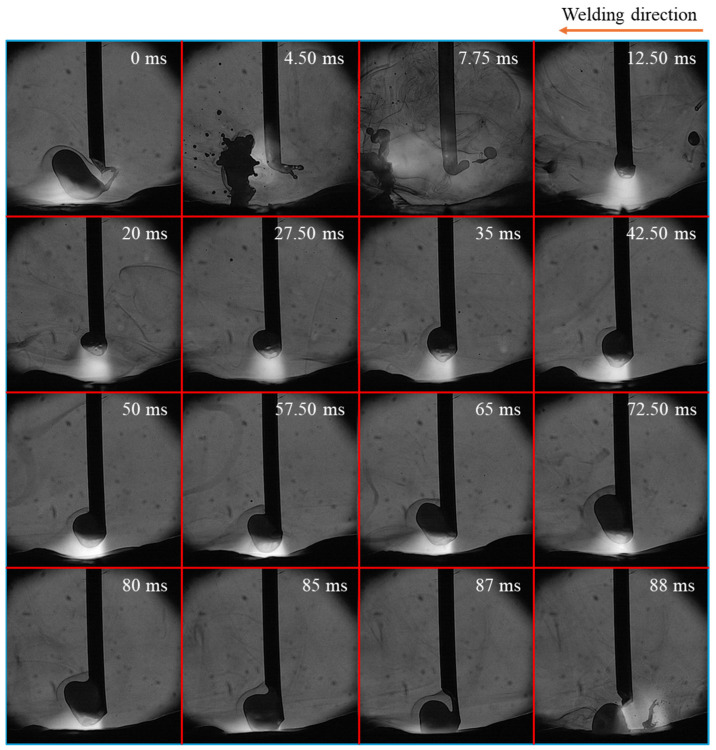
Time-sequential images of a droplet in one cycle of metal transfer in conventional CO_2_ arc welding.

Figure 3 shows the time-sequential images of a droplet in one cycle of metal transfer in conventional CO_2_ arc welding, considered from the moment of one detachment to the next one. The repelled transfer mode was observed, characterized by a large droplet being pushed upward and positioned on one side of the wire’s axis, along with the appearance of a long tail, which was formed at the rear of the droplet just before the detachment. The tail is defined as a long molten metal column including a largely extended neck before detachment and a long molten metal column after detachment. During the 88 ms detachment process, this tail was formed at the upper end of the molten metal at around 1 ms before the detachment. The detachment occurred when the tail became sufficiently thin. The tail oscillated and collided with the detached droplet, causing it to explode. This exploded droplet was propelled away from the weld pool (from 4.50 ms to 7.75 ms), thereby forming the large spatter.

**Figure 4:**
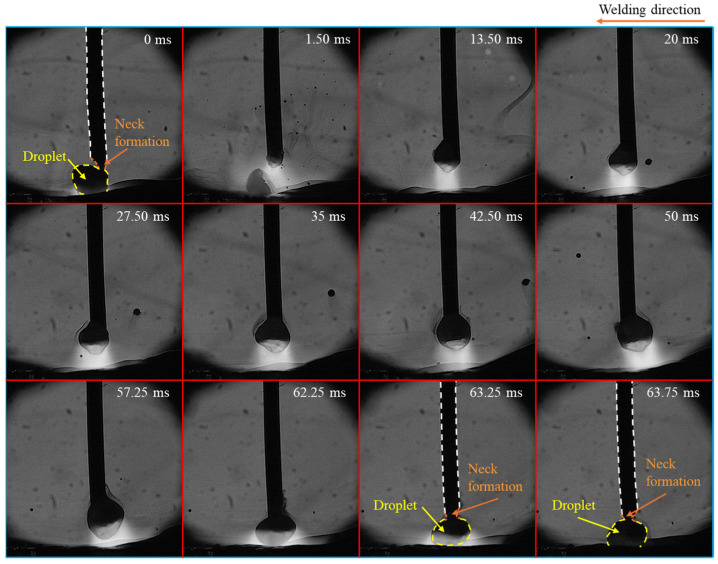
Time-sequential images of a droplet in one cycle of metal transfer in CO_2_ arc welding at MFD of 2 mT.

Figure 4 illustrates time-sequential images of a droplet in one cycle of metal transfer in CO_2_ arc welding at MFD of 2 mT. In this case, the metal transfer mode transitioned to globular transfer. The droplet exhibited an oval shape, with the upper end smaller than the bulging middle (teardrop shape), and remained aligned with the center of the wire axis during its growth (from 35 ms to 57.25 ms). The molten metal was observed rotating counterclockwise when seen from the top. Unlike in conventional CO_2_ arc welding, a neck formed near the wire tip instead of a long tail (at 0 ms, 63.25 ms, and 63.75 ms). And the duration from the formation of the neck to the separation of the droplet was very short, lasting about 1.50 ms (from 62.25 ms to 63.75 ms). This result implies that the detachment force works properly.

**Figure 5:**
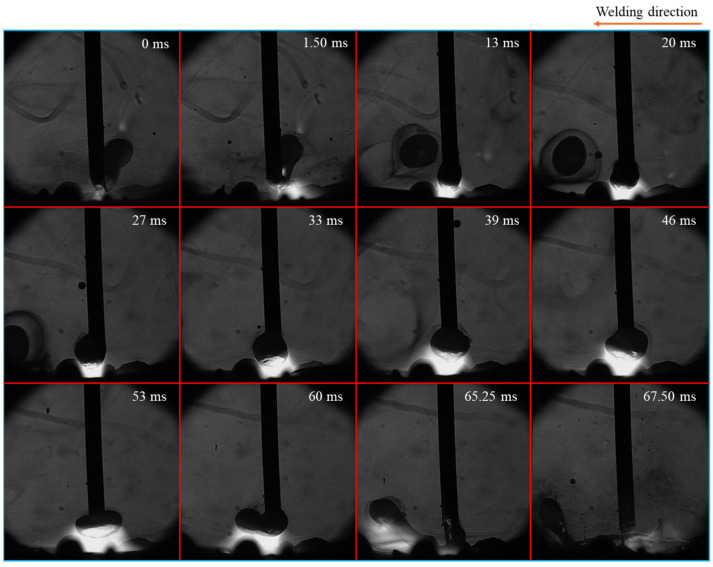
Time-sequential images of a droplet in one cycle of metal transfer in CO_2_ arc welding at MFD of 4 mT.

Figure 5 depicts time-sequential images of a droplet in one cycle of metal transfer in CO_2_ arc welding at MFD of 4 mT. The repelled droplet was observed to rotate around the wire, indicating a transition in the metal transfer mode from globular to rotating repelled transfer, which was a unique transfer mode in CO_2_ arc welding applying large MFD. During the middle stage of droplet growth process (from 33 ms to 46 ms), the metal transfer behavior was similar to that observed at 2 mT. However, after that, the droplet became elongated. From 53 ms to 60 ms, the molten metal column rotated around the wire axis, with its center of rotation at the wire tip. In detail, at 53 ms, this column was positioned behind the wire and perpendicular to the plane of the paper before gradually rotating to the wire’s left horizontal position (at 60 ms). As this column grew longer, its middle part sagged and eventually came into contact with the weld pool (at 65.25 ms). This contact resulted in the column’s separation through an explosive event (at 67.50 ms). Additionally, detached molten metals were observed outside the weld pool during the periods from 13 ms to 27 ms and at 67.50 ms, which were identified as spatters.

#### 3.1.2. Arc Phenomenon

The arc phenomenon was also observed (refer to Figure 3, Figure 4 and Figure 5), although the primary purpose focused on metal transfer behavior. The arc attachment consistently appeared under the bottom of the droplet, regardless of the MFD. The length of the arc column decreased with the increase in MFD. The arc shape differed notably between conventional CO_2_ arc welding and cases with the LMF. Under the influence of the LMF, the upper end of the arc contracted, while the lower end near the weld pool expanded. Additionally, the arc rotated counterclockwise, similarly to the molten metal. The rotation speed of the arc increased with higher MFDs.

#### 3.1.3. Spatter Formation

Spatter formation—defined as melted material leaving either from a droplet or the welding pool and attaching to the workpiece surface [24]—is an integral part of the metal transfer process in welding. Therefore, spatter formation was evaluated by analyzing metal transfer behavior in CO_2_ arc welding both without and with the application of the LMF. The variation in spatter as a function of MFD was evaluated by a qualitative method—the total 4 s duration (16,000 frames) of each video clip was checked carefully to assess the spatter formation occurrence under the effect of LMF.

**Figure 6:**
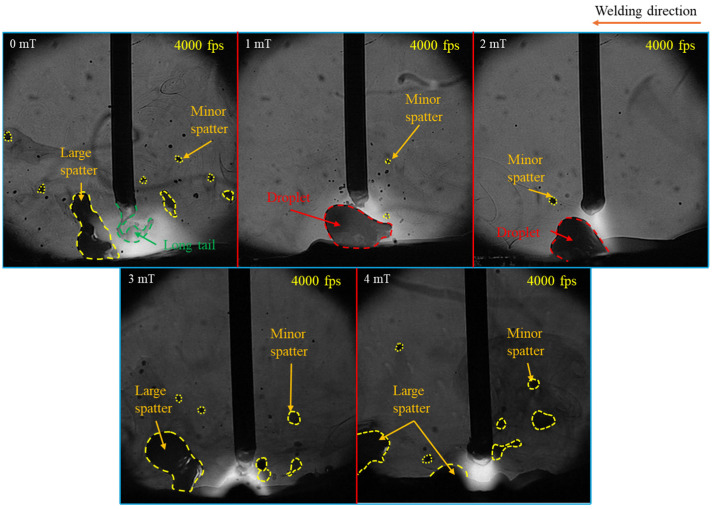
Typical images of spatter formation in CO_2_ arc welding under different MFDs.

Figure 6 shows typical images of spatter formation in CO_2_ arc welding under different MFDs. The analysis revealed that the quantity, size, and spatial range of spatter significantly decreased as the MFD increased from 0 to 2 mT. However, at MFD of 3 mT and 4 mT, spatter formation increased notably, with larger spattering droplets being produced.

**Figure 7:**
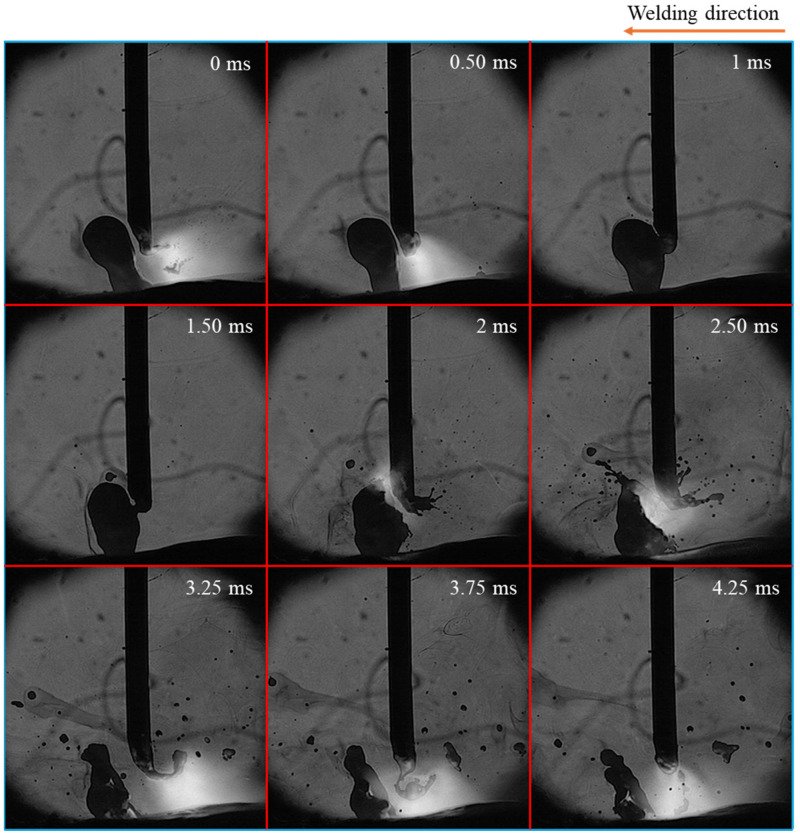
Time-sequential images of spatter formation in conventional CO_2_ arc welding.

Figure 7 illustrates the time-sequential images of spatter formation in conventional CO_2_ arc welding. It indicated that spatter formation and the motion of the long tail were positively correlated. Spatter formation occurred in two stages: at the moment of droplet detachment and seconds after that due to the movement of the tail.

In the first stage (at 0 ms), the tail moved in the direction opposite to the droplet, leading to the formation and detachment of smaller droplets, which resulted in spattering. In the second stage, the tail moved back and touched the droplet (at 1 ms), causing an explosion (at 2 ms). This explosion produced numerous smaller droplets of varying sizes, which spattered in all directions. Larger molten metal droplets from the explosion—large spatters—also moved away from the weld pool (at 4.25 ms).

**Figure 8:**
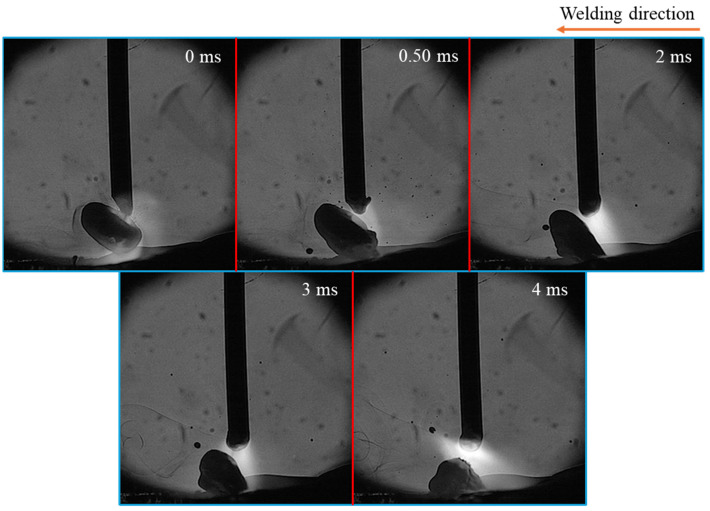
Time-sequential images of spatter formation at MFD of 2 mT.

Figure 8 illustrates time-sequential images of spatter formation at MFD of 2 mT during 4 ms from the droplet detachment. The presence of a short neck allowed the droplet to detach smoothly with very little spatter, and no long tail—as observed in the case without LMF—was formed after detachment.

**Figure 9:**
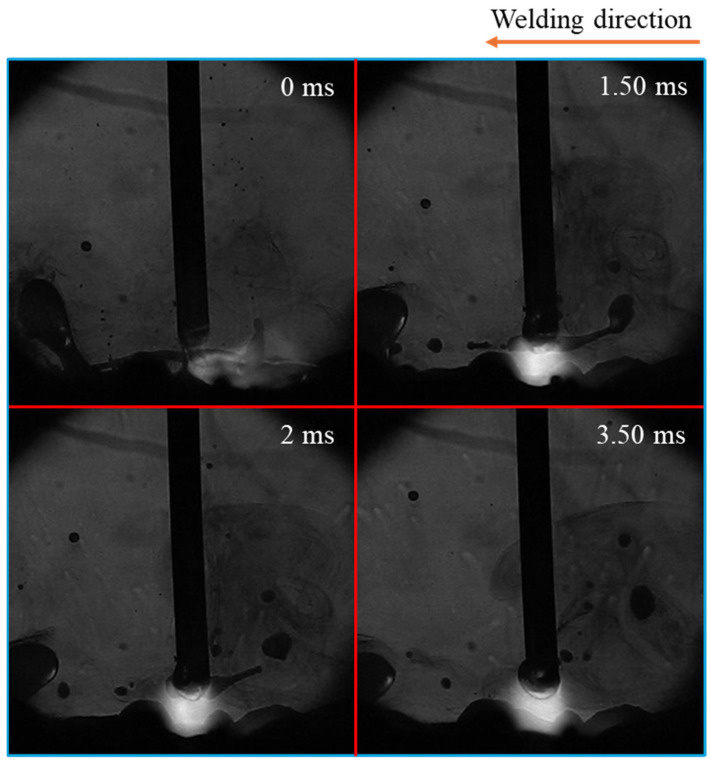
Time-sequential images of spatter formation at MFD of 4 mT.

Figure 9 illustrates time-sequential images of spatter formation at MFD of 4 mT. Unlike the case for 2 mT, a significant amount of spatter reappeared within a very short duration (4 ms), immediately after droplet detachment. The middle part of a molten metal column sagged and touched the weld pool, leading to an explosion of molten column. When this explosion occurred, numerous molten metal droplets spattered in all directions. Similarly to the conventional CO_2_ arc welding case, large droplets from the explosion were observed outside the weld pool.

### 3.2. Simulation Model and Results

#### 3.2.1. Simulation Model

A coupled arc–droplet numerical simulation model based on the Computational Fluid Dynamics (CFD) method, which was introduced in detail by the previous works [25,26,27], was also established to provide more information on the metal transfer behavior and arc characteristics under the effect of the LMF with MFD of 0, 2 mT and 4 mT in CO_2_ arc welding. The governing equations, including VOF (Volume of Fluid), mass, momentum, energy, and metal vapor transport, are shown below.

Fluid volume fraction:

(1) $$\frac{\partial F_{m}}{\partial t}+\nabla \cdot \left(F_{m}v_{m}\right)=0$$

Mass continuity:

(2) $$\frac{\partial \rho_{i}}{\partial t}+\nabla \cdot \left(\rho_{i}v_{i}\right)=0$$

Momentum conservation:

(3) $$\frac{\partial \left(\rho_{i}v_{i}\right)}{\partial t}+\nabla \cdot \left(\rho_{i}v_{i}v_{i}\right)=-\nabla P+\nabla \cdot \tau +j\times B+S_{u}$$

Energy conservation:

(4) $$\frac{\partial \left(\rho_{i}h_{i}F_{i}\right)}{\partial t}+\nabla \cdot \left(\rho_{i}v_{i}h_{i}F_{i}\right)=\nabla \cdot \left(k_{i}\nabla T_{i}\right)+\frac{j^{2}}{\sigma_{i}}F_{i}+S_{T}$$

Metal vapor mass conservation:

(5) $$\frac{\partial \left(\rho_{g}{CF}_{g}\right)}{\partial t}+\nabla \cdot \left(\rho_{g}{Cv}_{g}F_{g}\right)=\nabla \cdot \left(D\nabla CF_{g}\right)+M_{vap}$$

where *F* is the volume fraction; *t* is the time; v is the velocity vector; i indicates gas or metal phase; ρ is the density; *P* is the static pressure; ***τ*** is the viscous shear tensor; ***j*** is the current density vector; ***B*** is the magnetic flux density vector; *h* is the specific enthalpy; *k* is the thermal conductivity; *T* is the temperature; *σ* is the electric conductivity; *C* is the mass fraction concentration of iron metal vapor; and *D* is the diffusion coefficient of iron metal vapor in pure CO_2_ shielding gas. $M_{vap}$ is the mass source of metal vapor.

The source term in the metal phase of the momentum conservation equation is

(6) $$S_{u}=\rho_{m}g+\mu_{g}\frac{\partial v_{g}}{\partial S}\cdot \left|\nabla F_{m}\right|+{\gamma k}_{cur}{\nabla F}_{m}$$

where *μ*_g_ is the gas phase dynamic viscosity; ***S*** is the tangential normal vector to the free surface; *γ* is the surface tension coefficient; and k_cur_ is the curvature.

The source terms *S*_T_ in the energy conservation equation between the two phases are different. In the gas phase, the net radiation efficiency *ε*_n_ of CO_2_-Fe mixture plasma is used:

(7) $$S_{T}={-4\pi \varepsilon }_{n}F_{g}\;$$

In the metal phase, heat conduction and electrode heating are included:

(8) $$S_{T}=\int_{T_{m}}^{T_{g}}k_{g}{dT}_{g}/\delta_{gm}\left|{\nabla F}_{m}\right|+\left|\frac{j}{e}\cdot {\nabla F}_{m}\right|\phi_{a}\;$$

where *δ*_gm_ is the thickness of the mixture region; *e* is the elementary charge; and *ϕ*_a_ is the work function of the anode material. The evaporating heat loss and input are not considered in this work, since the temperature boundary condition of the ground wall is a constant value of 3000 K, which will lead to much more metal vapor being generated and then impede the current pass.

**Figure 10:**
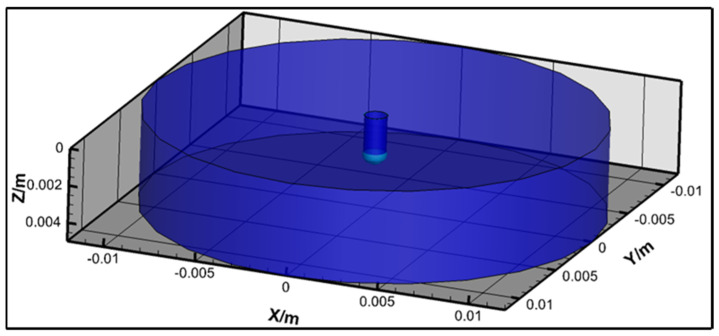
Schematic diagram of the 3D calculation domain.

Figure 10 shows a schematic diagram of the 3D calculation domain with a column with a diameter of 24 mm. The mild steel wire had an initial length of 2 mm and a diameter of 1.2 mm, with an arc length of 3 mm. The metal and gas inlets were located at the top of the domain, while the sides serve as gas pressure outlets. The bottom boundary functions as the ground wall, set to an electric potential (Φ) of 0 and a temperature of 3000 K, as specified.

For the metal vapor including only iron species in the arc plasma, the diffusion coefficient *D* based on the second viscosity approximation method was applied according to Murphy [28]. The thermophysical transport properties and net emission coefficients of the CO_2_-Fe mixture plasma were referenced from [12,28,29,30]. The physical properties of molten metal and welding parameters are listed in Table 3 and Table 4. The LMF was assumed to be uniform and was directed downward.

#### 3.2.2. Simulation Results

**Figure 11:**
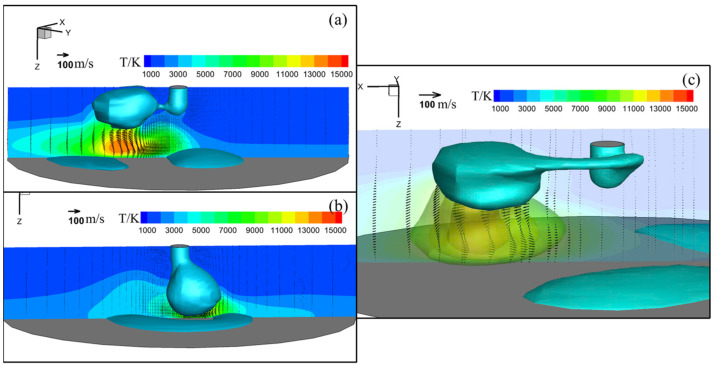
Arc temperature and velocity fields for (**a**) conventional CO_2_ arc welding and for MFDs of (**b**) 2 mT and (**c**) 4 mT in gas phase immediately before detachment.

Figure 11 shows arc temperature and velocity fields for (a) conventional CO_2_ arc welding and MFDs of (b) 2 mT and (c) 4 mT in gas phase immediately before detachment. The metal transfer modes and their characteristics were consistent with the experimental results. The maximal arc temperature immediately before detachment varied depending on the application of LMF and the magnitude of MFD. Without LMF, the maximal arc temperature reached approximately 16,000 K, with the highest-temperature region located under the offset droplet bottom due to the outward arc flow with maximal value of 80 m$\cdot s^{-1}$. When an MFD of 2 mT was applied, the maximal arc temperature decreased to around 15,000 K, and the highest region of temperature shifted to the region under the droplet bottom around the wire axis, differing significantly from the no-LMF condition. While the maximal arc velocity was the same 80 m$\cdot s^{-1}$ as that without the LMF. However, under an MFD of 4 mT, the maximal arc temperature returned to about 16,000 K, located directly under the droplet bottom, and the maximal arc velocity reached 130 m$\cdot s^{-1}$ at the moment of detachment.

**Figure 12:**
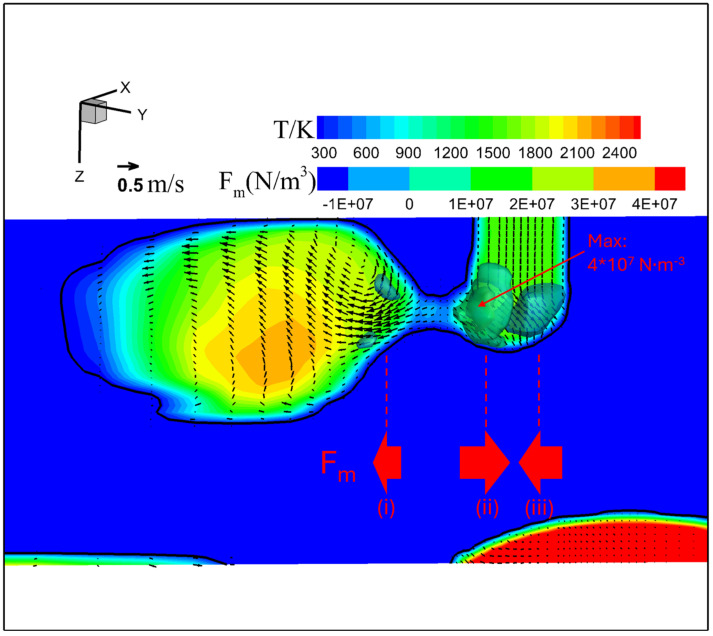
Temperature, velocity, and Lorentz force fields in metal phase immediately before droplet detachment for conventional CO_2_ arc welding.

Figure 12 shows the temperature, velocity, and Lorentz force (a horizontal component parallel to the presented plane depicted as 3D iso-surfaces) fields in metal phase immediately before droplet detachment for conventional CO_2_ arc welding. The maximal temperature, located in the lower region, was approximately 2200 K. The molten metal within the droplet and at the wire tip flowed in opposite directions around the neck. The velocity was found to be the highest either near the wire tip or at the upper part of the droplet. Although the Lorentz forces were concentrated around both ends of the neck, a significant imbalance was seen among them. The maximal force in the direction from left to right (ii) reached 4 × 10^7^ N·m^−3^—10 times larger than that in the opposite direction (i) and (iii). The significant force imbalance led to explosion of the tail and spatter formation, marked by a powerful outward flow with a maximum velocity of approximately 0.80 m·s^−1^ near the wire tip.

**Figure 13:**
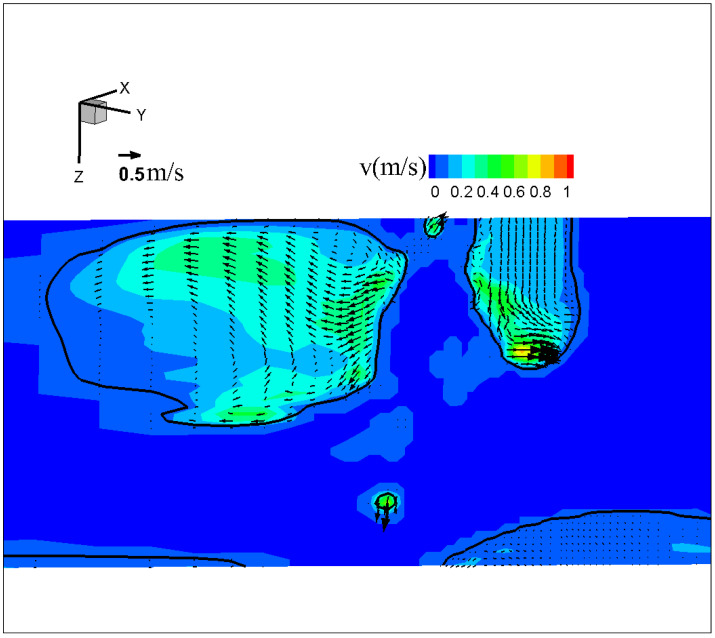
Molten metal velocity and spatter formation for conventional CO_2_ arc welding right after detachment.

Figure 13 shows the spatter formation and molten metal velocity right after droplet detachment. It indicated that some spatters appeared when the droplet detached. The high-velocity region of molten metal was concentrated horizontally at the wire tip, from left to right.

**Figure 14:**
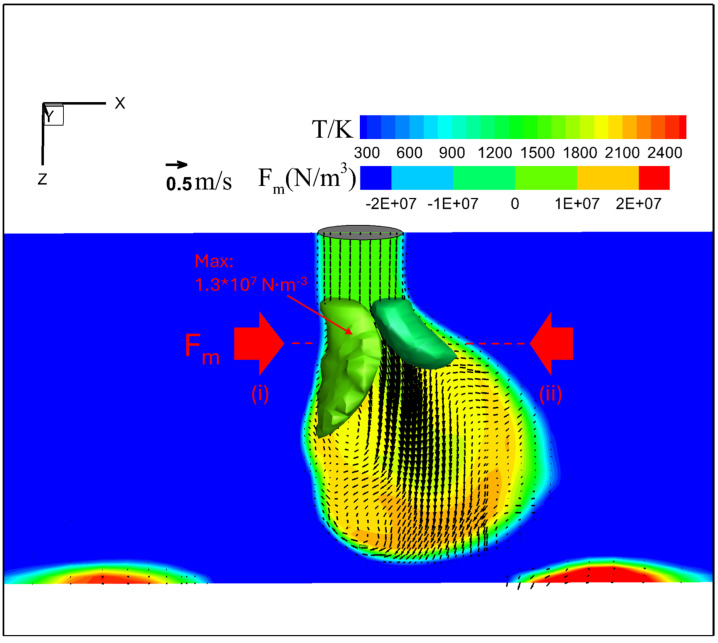
Temperature, velocity, and Lorentz force fields in metal phase immediately before droplet detachment for an MDF of 2 mT.

Figure 14 shows the temperature, velocity, and Lorentz force (a horizontal component parallel to the presented plane depicted as 3D iso-surfaces) fields in metal phase immediately before droplet detachment for an MDF of 2 mT. The temperature reached a maximum value of about 2400 K under the droplet. The Lorentz force acted symmetrically on both sides of the neck. The maximal Lorentz force in the direction from left to right (i) reduced significantly to approximately 1.3 × 10^7^ N·m^−3^, almost matching the force in the opposite direction (ii), causing the downward flow along with the droplet central axis with the maximal velocity to be 0.70 m$\cdot s^{-1}$. This balanced force field shifted the position of droplet formation from off-axis to on-axis.

Additionally, the spin force—the rotational Lorentz force to the wire axis expressed by cross-product of the arc current density and LMF vectors—caused both the arc and molten metal inside the droplet to rotate counterclockwise.

**Figure 15:**
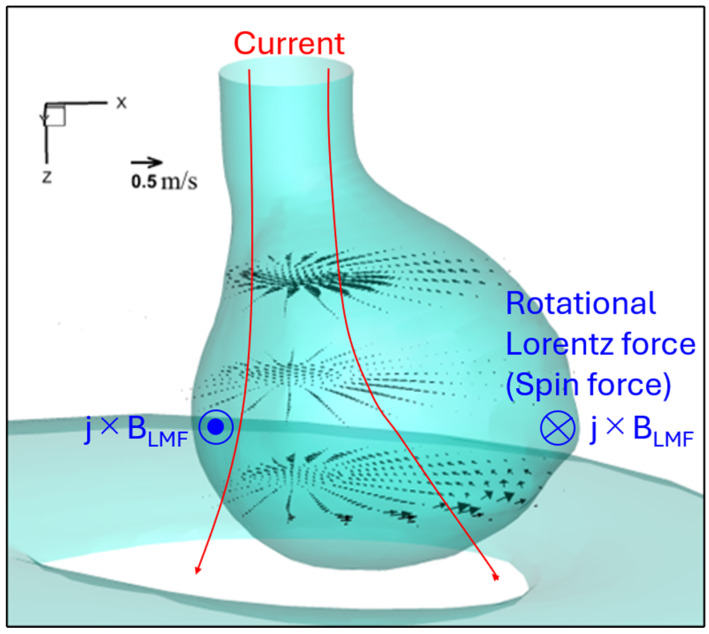
Horizontal velocity vector field on cross sections at z = 0.002 mm, z = 0.003 mm, and z = 0.004 mm for MFD of 2 mT at immediately before detachment.

Figure 15 depicts the horizontal velocity vector field on cross sections at z = 0.002 mm, z = 0.003 mm, and z = 0.004 mm for MFD of 2 mT at immediately before detachment. Notably, the maximum rotating velocity decreased from 0.26 m·s⁻¹ to 0.11 m·s⁻¹ when z increased from 0.002 mm to 0.003 mm, and then increased again to 0.50 m·s⁻¹ at z = 0.004 mm. Furthermore, the radial position where the maximum rotating velocity appeared shifted from the center of the droplet to its edge with the change in z from 0.002 mm to 0.004 mm.

**Figure 16:**
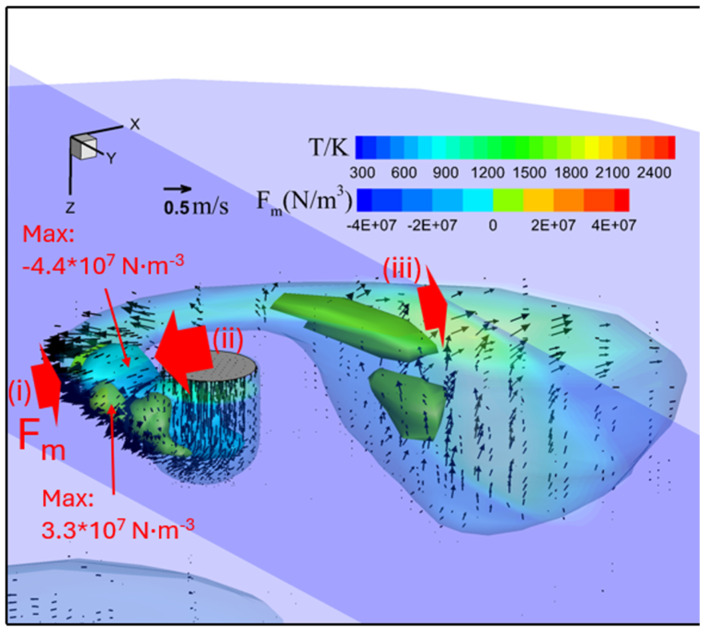
Temperature, velocity, and Lorentz force fields in metal phase immediately before droplet detachment for an MDF of 4 mT.

Figure 16 shows the temperature, velocity, and Lorentz force (x component depicted as 3D iso-surfaces) fields in metal phase immediately before droplet detachment for an MDF of 4 mT. In this case, the maximal temperature, now located in the upper region, was again approximately 2200 K. The molten metal column rotated in a counterclockwise direction around the wire, with the center of rotation located at the wire tip. The velocity of the molten metal was highest at the upper part of molten column near the wire tip, with the maximal velocity of 2 m$\cdot s^{-1}$ aligning with the direction of rotation.

The Lorentz force field presents two positive-force regions and one negative-force region. One of the positive regions near the wire tip (i) exhibited a maximal magnitude of 3 × 10^7^ N·m^−3^, while the negative region (ii) reached −4.4 × 10^7^ N·m^−3^. The molten metal column rotated and flowed outward due to these forces. The other positive region (iii), near the large droplet, lacked an opposing force, leading to upward and outward molten metal flow due to the constricted current at the bottom of the droplet.

**Figure 17:**
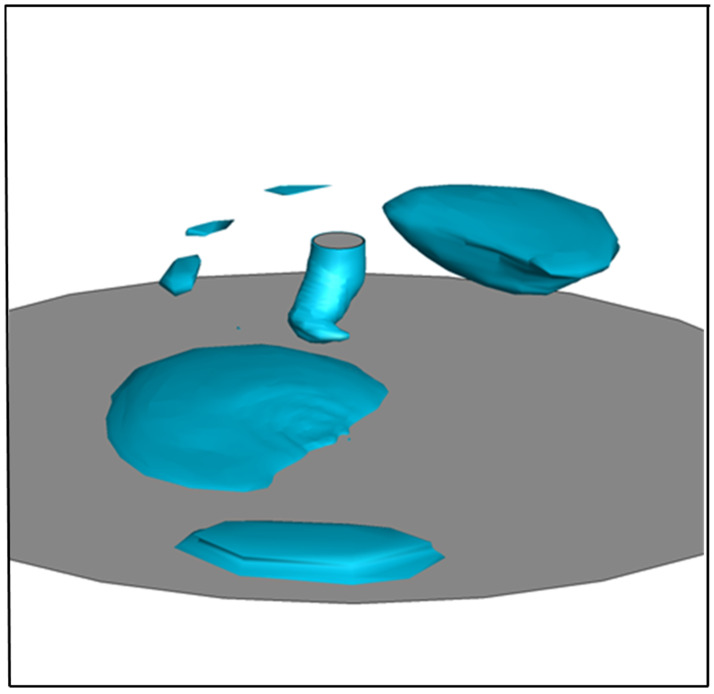
Spatter formation right after detachment for MFD of 4 mT.

The intense rotational motion from the LMF generated significantly more spatter compared to the 2 mT case, as shown in Figure 17.

## 4. Discussion

The results both negate and support the initial hypotheses. While it was predicted that applying LMF in CO_2_ arc welding would stabilize the arc and metal transfer while reducing spatter, this was not true in all cases. At the low MFD of 2 mT, globular transfer occurred with a decrease in spatter formation. However, at the high MFD of 4 mT, a transition to rotating repelled transfer was observed, along with a reappearance of spatter. To better understand the effects of the LMF on metal transfer behavior and spatter formation, this section highlights two key points: the transition in metal transfer behavior and the mechanism responsible for reducing spatter formation.

Firstly, the effects of LMF on metal transfer behavior are considered. The observed metal transfer behavior was governed by two key factors: the properties of the arc plasma and the forces acting on the droplet [4].

**Figure 18:**
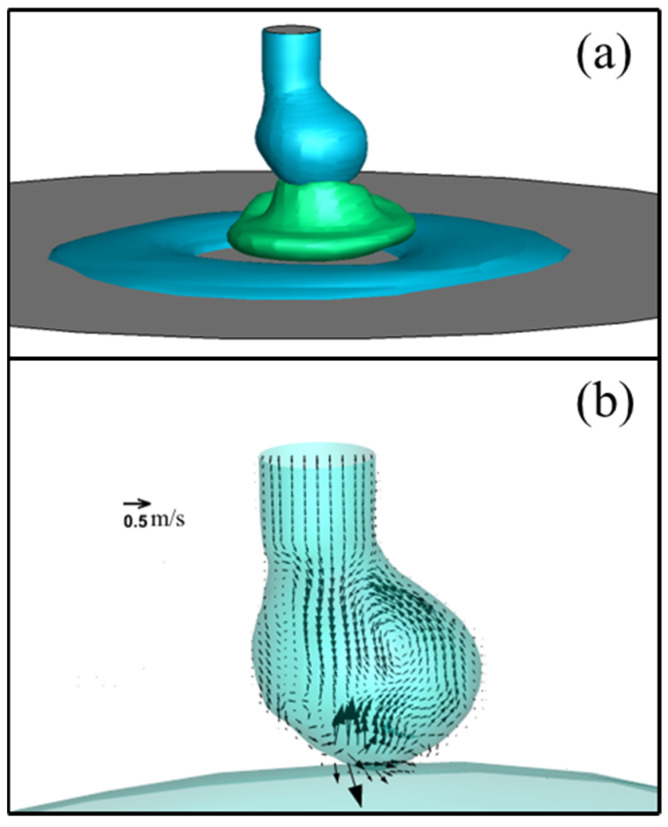
(**a**) Arc shape and (**b**) molten metal velocity field during the middle stage of the metal transfer process in conventional CO_2_ arc welding.

Figure 18 depicts the arc shape and molten metal velocity field during the middle stage of the metal transfer process, in which the droplet is not yet repelled in conventional CO_2_ arc welding. In a case without LMF, Miao et al. reported that the arc velocity was symmetrically distributed to the arc’s central axis, flowing radially outward from the center to the edge on the base metal surface [12], which was consistent with our results. In our results, the high-temperature region of the arc was also located directly under the bottom of the droplet. Due to the constricted arc caused by a high specific heat of the CO_2_ arc, the arc temperature increased at the center, and the current predominantly flowed through the arc center under the bottom of the droplet. The current constriction generated high arc pressure under the bottom of the droplet, stemming the strong downward molten metal flow along the center to induce the upward flow on the side to form an eddy inside the droplet. Consequently, the droplet was pushed upward, resulting in repelled transfer.

Under an MFD of 2 mT, the centrifugal effect caused by the rotational motion of the molten metal inside the droplet is considered to keep the droplet position at the center, as shown in Figure 15. This centrifugal effect is thought to also cause the droplet to adopt an oval shape (teardrop shape). As shown in Figure 14, the calculated droplet temperature is higher than that for conventional CO_2_ arc welding, lowering the arc temperature around the center due to the stronger radiation loss by the denser metal vapor plasma that forms through evaporation from the droplet. The reduction in arc temperature lowers the current flow through the arc center due to a decrease in the electrical conductivity, decreasing the arc pressure directly under the droplet. Instead, a larger part of the current is considered to flow through the surrounding shielding gas plasma. The decrease in the arc pressure could be one of the factors preventing the transition to repelled transfer. According to these factors, the droplet could easily grow toward the welding pool rather than being repelled. Additionally, the high current density in this narrow upper part created a strong Lorentz force acting on the upper end of the droplet, forming a neck. As a result, the globular transfer occurred instead of the repelled transfer.

The mechanism to cause the rotating repelled transfer under an MDF of 4 mT was elucidated. In this case, the arc temperature at the center was the same as that in conventional CO_2_ arc welding, leading to an increase in pressure under the bottom of the droplet. This pressure prevented the molten metal from moving toward the weld pool. Combined with the very high velocity of the arc and molten metal flowing from the wire tip into the droplet, a large droplet was pushed upward to one side and elongated, causing the molten metal to enter an asymmetrical state. At this point, under a very strong centrifugal effect, the molten metal column itself rotated around the wire axis and became longer, rather than the rotational motion of the molten metal inside the droplet for 2 mT case.

Secondly, the mechanism of spatter formation was examined. Ogino et al. explained spatter formation in the repelled transfer mode through a simulation model that captured the asymmetric behavior of the molten droplet in CO_2_ arc welding [31]. In our experiments, we identified two stages of spatter formation during the repelled transfer: the first stage involves small droplets detaching from the curved long tail when the droplet separated, while the second stage is related to the tail moving back and colliding with the detached droplet, causing an explosion and generating more spatter. Their report [31] suggests that the strong Lorentz force acting on the tail causes instability in the droplet, leading to the detachment of small droplets and spatter formation, which corresponded to the first stage of our findings.

**Figure 19:**
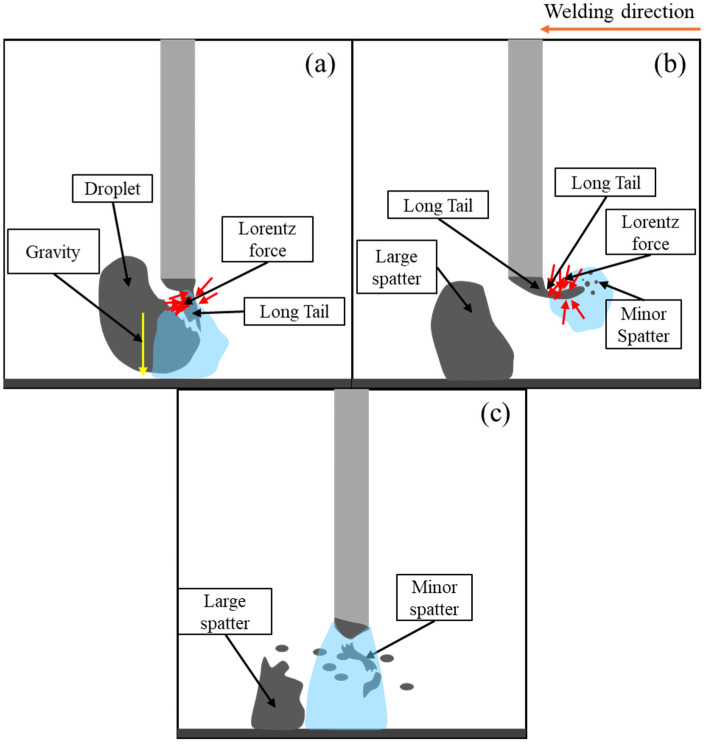
Schematic of spatter formation in conventional CO_2_ arc welding: (**a**) moment of detachment; (**b**) first stage of spatter formation; and (**c**) second stage of spatter formation.

Figure 19 illustrates two stages of spatter formation in the repelled transfer mode according to our suggestion. In the first stage, due to the formation of the curved long tail, the magnetic fields on either side of it differed significantly, creating an imbalance in the Lorentz force in opposite directions. This imbalanced force strongly pushes both the tail and droplet from the side with a larger magnetic field to that with a smaller magnetic field, generating spatters during droplet detachment (Figure 19a,b). In the second stage, the strong Lorentz force elevated the long tail to a high position near the wire tip. At this position, the difference in magnetic field was formed in the opposite direction to that in the first stage, causing another imbalance in the Lorentz force. This made the molten tail move back toward the droplet, thus coming into contact with it. This contact led to a short circuit, causing an explosion of both the droplet and the tail (Figure 19c). The droplet moved out of the weld pool, resulting in a large spatter, accompanied by smaller spatters from the tail.

Yamazaki et al. successfully reduced spatter in CO_2_ gas-shielding arc welding by regulating globular transfer with a specialized pulsed current waveform [2]. While the customized welding power source can be expensive, our approach demonstrated that applying a low-cost external magnetic field device to a conventional GMAW power source offered a more affordable solution. In our study, a stable globular transfer was achieved through the continuous application of a low MFD, in contrast to Yamakazi’s method, which required sophisticated control of a pulsed current waveform at two critical stages—droplet separation and droplet formation—to avoid short-circuiting and deformation.

**Figure 20:**
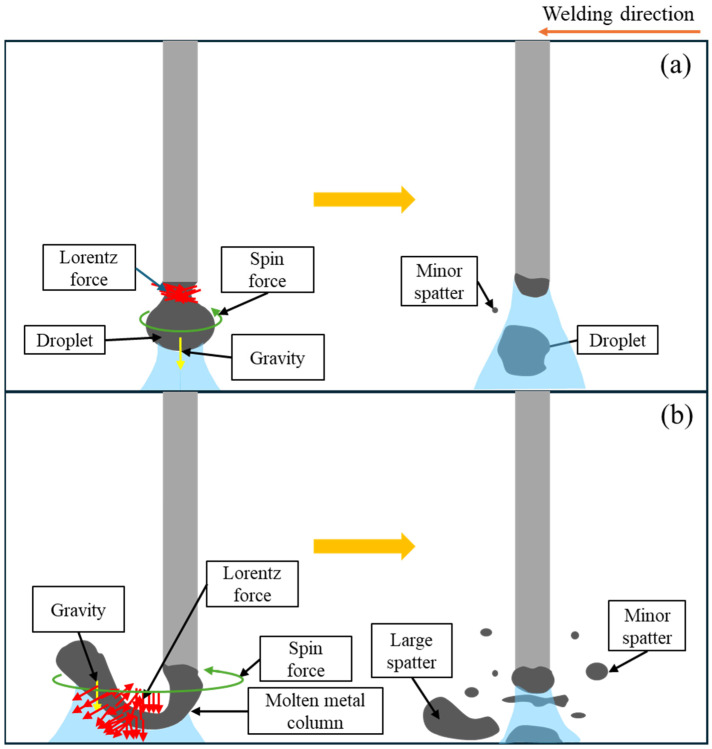
Schematic of forces acting on a droplet just before detachment and spatter generation under magnetic flux densities of (**a**) 2 mT and (**b**) 4 mT.

Interestingly, our findings revealed that there is an optimal MFD for suppressing spatter formation. The mechanisms of spatter formation at MFDs of 2 mT and 4 mT are illustrated in Figure 20.

At a lower MFD case, the neck formation played a significant role. When the neck formed symmetrically to the wire axis, most of the current flowed through this area (Figure 20a), concentrating the Lorentz force to this region. Due to the balance of Lorentz forces in opposite directions, the neck gradually became thinner, leading to smooth droplet detachment. Since the detachment occurred so close to the wire tip, no molten tail was formed, resulting in minimal spatter.

Under the effects of an MFD of 4 mT, a long molten column formed and rotated at a high speed, causing the column to take on a spiral shape. The arc was confined to a part of the column near the wire tip. A strong Lorentz force acting on the molten metal near the wire tip pushed this part downward, causing it to touch the weld pool (Figure 20b). This resulted in an explosion of the molten part, generating numerous spatters. Due to the high centrifugal force, the larger portion of the molten metal column was ejected from the weld pool, which is a significant contributor to spatter issues in welding.

For the first time, we have revealed the transition of metal transfer from the repelled transfer to the globular transfer and then to the rotating repelled transfer, along with their associated spatter formation mechanisms, in a CO_2_ arc welding both with and without the application of LMF. Our approach of applying an optimal MFD, ranging from 1 mT to 2 mT, to control a stable globular transfer and reduce spatter is highly significant for industrial applications. It would also be interesting to explore the potential of using machine learning to control metal transfer behavior in this process when LMF is applied, further minimizing spatter. Additionally, an equally intriguing direction is to investigate the later stages of this study, focusing on the behavior of the weld pool and the characteristics of the weld bead under the influence of LMF.

## 5. Conclusions

This study investigated the effects of LMF on metal transfer behavior and spatter formation in CO_2_ arc welding through experiments and simulations. The results showed that increasing MFD from 0 to 2 mT shifted the transfer mode from the repelled transfer to the globular transfer, while higher MFDs (3–4 mT) induced the rotating repelled transfer. The globular transfer at 2 mT is considered to be primarily produced by the centrifugal effect due to the rotational motion of the molten metal inside the droplet, which is caused by the Lorentz force affected by LMF. The higher droplet temperature in this condition also contributed to forming this transfer mode, preventing the formation of repelled transfer through a decrease in the arc pressure. Contrastingly, in the higher MFDs, the droplet temperature decreased, increasing the arc pressure, which lifted the droplet up. Furthermore, the very strong centrifugal effect rotated the molten metal column around the wire axis to induce the rotating repelled transfer. The spatter formation was found to occur with the two-stage motion of the curved long tail without LMF and at 4 mT, and also with the exploding molten metal column at 4 mT, due to an imbalance of the Lorentz force acting on the molten metal. On the other hand, the neck formation facilitated smooth droplet detachment without forming the curved long tail at 2 mT, significantly reducing spatter. These findings offer valuable insights for optimizing welding quality and efficiency by stabilizing globular transfer under an optimal LMF.

## Figures and Tables

**Table 1:** Composition of base metal and filler metal [23].

Elements	C	Si	Mn	P	S	Ti + Zr	Fe
Base material	0.26	0.40	-	0.04	0.05	-	Bal.
Filler material	0.08	0.51	1.10	0.01	0.01	0.05	Bal.

**Table 2:** Welding conditions.

Item	Value
Welding current	250 A
Arc voltage	33.5 V–34.0 V
Welding velocity	$5\;\mathrm{mm}\cdot s^{-1}$
Shielding gas	$100\%\;{\mathrm{CO}}_{2};\;20\;L\cdot {min}^{-1}$
CTWD	20 mm
Magnetic flux density	0–4 mT

**Table 3:** Physical properties of gas and metal phases.

Nomenclature	Symbol	Unit	Gas Phase	Metal Phase
Density	ρ	$Kg\cdot m^{-3}$	[28]	7200
Dynamic viscosity	μ	$Kg\cdot m^{-1}\cdot s^{-1}$	[28]	0.006
Specific heat	$c_{o}$	$J{\cdot kg}^{-1}{\cdot K}^{-1}$	[28]	780
Thermal conductivity	k	$W\cdot m^{-1}\cdot K^{-1}$	[28]	22
Electrical conductivity	σ	$S\cdot m^{-1}$	[28]	$7.7\times 10^{5}$
Net emission coefficient	$\varepsilon_{n}$	$W\cdot m^{-3}\cdot {sr}^{-1}$	[28]	-
Work function	${ø}_{a}$	eV	-	4.5
Solidus temperature	$T_{s}$	K	-	1750
Liquidus temperature	$T_{1}$	K	-	1800
Vaporization temperature	$T_{v}$	K	-	3050
Surface tension coefficient	γ	$N\cdot m^{-1}$	-	0.9

**Table 4:** Welding parameters.

Nomenclature	Value
Current	DC 250 A
Wire diameter	1.2 mm
Wire feed rate	$0.15\;m\cdot s^{-1}$
Arc length	3 mm
CO_2_ shielding gas flow rate	$20\;L\cdot {min}^{-1}$
Magnetic flux density	2 mT and 4 mT

## Data Availability

The original contributions presented in this study are included in the article. Further inquiries can be directed to the corresponding authors.
